# Erdheim–Chester Disease With Multiorgan Involvement, Following Polycythemia Vera: A Case Report

**DOI:** 10.1097/MD.0000000000003697

**Published:** 2016-05-20

**Authors:** Alessandra Iurlo, Lorenzo Dagna, Daniele Cattaneo, Nicola Orofino, Paola Bianchi, Giulio Cavalli, Claudio Doglioni, Umberto Gianelli, Agostino Cortelezzi

**Affiliations:** From the Oncohematology Division, IRCCS Ca’ Granda - Maggiore Policlinico Hospital Foundation, and University of Milan (AI, DC, NO, PB, AC); Oncohematology Unit of the Elderly, IRCCS Ca’ Granda – Maggiore Policlinico Hospital Foundation (AI); Unit of Medicine and Clinical Immunology, IRCCS San Raffaele Scientific Institute, Vita-Salute San Raffaele University (LD, GC); Unit of Pathology, IRCCS San Raffaele Scientific Institute, Vita-Salute San Raffaele University (CD); and Hematopathology Service, Division of Pathology, Department of Pathophysiology and Transplantation, University of Milan and IRCCS Ca’ Granda – Maggiore Policlinico Hospital Foundation, Milan, Italy (UG).

## Abstract

Erdheim–Chester disease is a rare form of non-Langerhans cell histiocytosis characterized by the migration and infiltration of lipid-laden CD68^+^, CD1a^−^ and S100^−^ histiocytes to various target organs, which leads to the disruption of physiological tissue architecture and reactive fibrosis, and thus impairs organ function.

We describe the first case of a patient with Erdheim–Chester disease with multiorgan involvement developed after 6 years from polycythemia vera diagnosis. During the follow-up, an abdominal ultrasound scan revealed the presence of dense, bilateral perinephric infiltration. A computed tomographic guided core biopsy was performed in order to identify the histological nature of this lesion, and a morphological analysis demonstrated the accumulation of foamy histiocytes surrounded by fibrosis. The *BRAF*V600E mutation was detected, and a diagnosis of Erdheim–Chester disease was made.

The extreme rarity of Erdheim–Chester disease strongly suggests the existence of potentially common element(s) that may have contributed to the pathogenesis of both disorders. Obviously, further studies are needed to clarify the mutual roles and effects of *JAK2* and *BRAF* mutations in this patient, as well as their possible therapeutic implications.

## INTRODUCTION

Erdheim–Chester disease (ECD) is a rare form of non-Langerhans cell histiocytosis characterized by the migration and infiltration of lipid-laden CD68^+^, CD1a^−^, and S100^−^ histiocytes to various target organs, which leads to the disruption of physiological tissue architecture and reactive fibrosis, and thus impairs organ function.^1,2^ It seems to have a slight male predominance, the majority of the patients are diagnosed between the ages of 40 and 70 years,^3^ and its severity ranges from mild to life-threatening depending on the site(s) of involvement.^4^

It has been found that ECD histiocytes express a pro-inflammatory network of cytokines and chemokines that is responsible for local activation and recruitment of other pathological histiocytes.^5^ They also bear activating mutations in the *MAPK*-activating pathway, of which *NRAS* and *PIK3CA* mutations have so far been described in only a few cases, but the *BRAF*V600E mutation seems to be present in most patients.^6,7^

On the basis of these findings, ECD is now considered to be a clonal disorder associated with the development of a local and systemic pro-inflammatory milieu that plays a crucial role in its pathogenesis and clinical manifestations.^8^

Associations between ECD and other hematological malignancies have been rarely reported,^9^ and there is no previously published case involving the coexistence of ECD and *BCR-ABL1*-negative myeloproliferative neoplasms (MPNs). Polycythemia vera (PV) is an MPN that is mainly characterized by increased red cell mass, frequently associated with leukocytosis and thrombocytosis.^10^ Sixty percent of PV patients are older than 60 years, and there is no difference in gender distribution.^11^ Almost all of the patients present the *JAK2*V617F mutation and, albeit to a lesser extent, mutations in the gene's exon 12. Furthermore, PV is typically associated with an increased risk of thrombosis and a long-term tendency to transform itself into post-PV myelofibrosis (15–20%)^12^ and/or acute myeloid leukemia (5–8%).^11^

We here describe for the first time the case of a patient with a previous diagnosis of PV who developed ECD with multiorgan involvement after 6 years of hematological follow-up.

## CASE REPORT

A 66-year-old man was admitted into our Hospital in November 2008 with splenomegaly, a high red cell count, and subnormal serum erythropoietin levels. Cytogenetic analysis showed a normal male karyotype. Molecular studies were negative for the *BCR-ABL1* rearrangement, but revealed the *JAK2*V617F mutation with an allele burden of 44.9%. A diagnosis of PV was made on the basis of a morphological bone marrow analysis according to the WHO 2008 criteria. The patient was started on aspirin, hydroxyurea, and phlebotomies, all of which were well tolerated.

Six years later, an abdominal ultrasound scan revealed the presence of dense bilateral perinephric infiltration, which was confirmed by a whole-body computed tomography (CT) scan, and a fluotine-18 fluordeoxyglucose positron emission tomography (F-FDG-PET) scan demonstrated increased glucose uptake in the medullary channels of both humeri and femurs. Morphological bone marrow analysis confirmed the previous diagnosis of PV and showed no evident change in the grading of bone marrow fibrosis (MF-0 according to the EUMNET consensus),^13^ whereas cytogenetic analysis revealed the following complex male karyotype: 47,XY,+9[2/20],47,XY,add(6)(p25),+9[2/20],46,XY[16/20].

**FIGURE 1:**
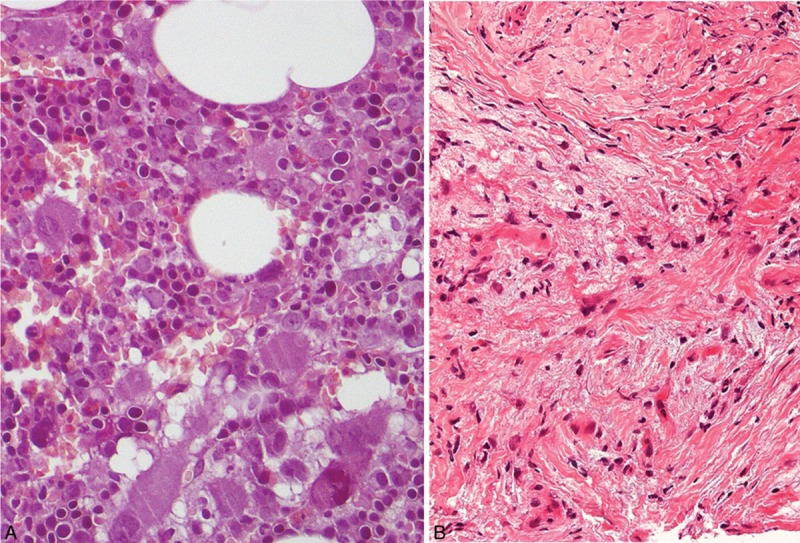
A, Morphological bone marrow analysis confirming the previous diagnosis of PV, with no evident change in bone marrow fibrosis or blast percentage, and no significant lymphoid infiltrate. B, CT-guided biopsy of the perinephric infiltrating lesion showing the accumulation of foamy histiocytes positive for CD68 and negative for the dendritic cell markers CD1a and S100, surrounded by fibrosis and a scanty lympho-monocytic infiltrate.

**FIGURE 2:**
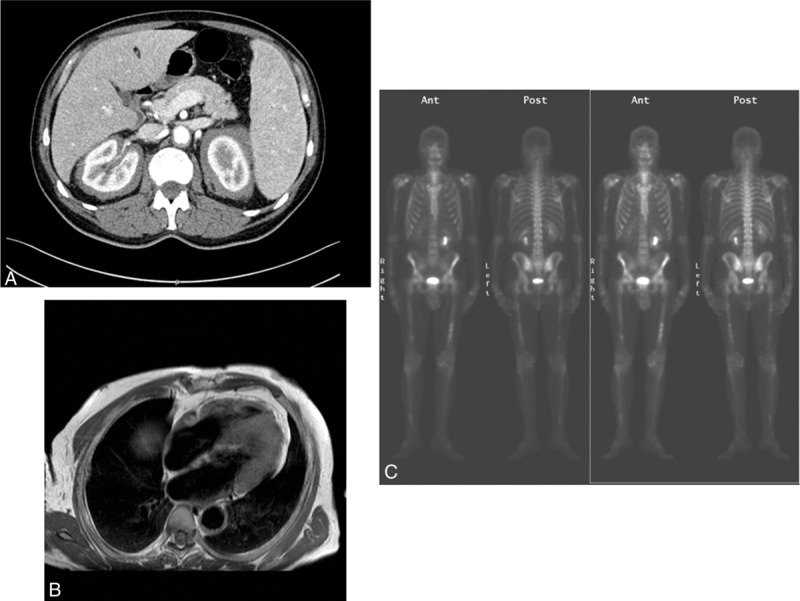
A, Contrast-enhanced abdominal CT scan showing solid vascularized tissue located in the retroperitoneal space surrounding the kidneys, ureters and aorta. B, Cardiac MRI showing a mass located in the right atrioventricular sulcus. C, Bone scan showing symmetrically irregular radiotracer uptake in the long bones of the limbs.

The histological nature of the perinephric infiltration was identified by means of a CT-guided core biopsy of the lesion. Morphological analysis demonstrated the accumulation of foamy histiocytes that were positive for CD68 and negative for the dendritic cell markers CD1a and S100, surrounded by fibrosis and a scanty lympho-monocytic infiltrate (Figure 1). The detection of the *BRAF*V600E mutation led to a diagnosis of ECD. Cardiac magnetic resonance imaging (MRI) revealed pseudo-tumoral infiltration of the atrioventricular wall,^14^ and a bone scan showed symmetrically irregular radiotracer uptake in the long bones of the limbs consistent with ECD (Figure 2)^2^; the findings of cerebral and pulmonary imaging were negative. In accordance with the most recent guidelines for the treatment of ECD,^1^ the patient started therapy with pegylated interferon (IFN)-alpha and, after 3 months of treatment, a new abdominal ultrasound scan revealed the complete resolution of the dense bilateral perinephric infiltration, and the normalization of the blood cell counts was achieved.

## DISCUSSION

ECD is an extremely rare hematological disorder, as only about 600 cases have been reported in the literature^2^; PV is also an infrequent disease, with an estimated annual incidence of 2/100,000 people.^10^ To the best of our knowledge, there is only 1 published report describing ECD in association with another hematological neoplasms: the case of a 14-year-old girl with pre-B cell acute lymphoblastic leukemia in remission who was subsequently diagnosed as having ECD.^9^ There are no previously published descriptions of the coexistence of ECD and PV or other *BCR-ABL1*-negative MPNs.

A critical step in our understanding of the exact pathogenic mechanism underlying PV was the discovery of the *JAK2*V617F-acquired activating somatic mutation in 2005.^15^ Unlike the other *BCR-ABL1*-negative MPNs, about 95% to 97% of all cases of PV are characterized by the presence of this mutation, which is not seen in patients with lymphoid neoplasms or reactive myeloproliferation, or in healthy volunteers. *JAK2*V617F is due to a somatic G to T mutation involving *JAK2* exon 14, and affects the noncatalytic “pseudo-kinase” domain by derailing its kinase regulatory activity. The constitutive activity of *JAK2* mainly leads to an excessive transcription of cell survival promoting, anti-apoptotic molecules, and an increased production of pro-inflammatory cytokines, such as interleukin (IL)-6, tumor necrosis factor alpha (TNF-α), IL-8, IL-2R, IL-12, and IL-15 by pathological megakaryocytes and monocytes.^16^ As *JAK2*V617F does not seem to be the disease-initiating event but probably defines an MPN subclone, the possibility of the independent emergence of multiple abnormal clones has recently been suggested, which challenges the prevailing view that an abnormal ancestral clone gives rise to mutually exclusive subclones.

Such genome instability may lead to a predisposition to acquire additional somatic mutations, including those of the *BRAF* gene,^17^ which is involved in the cell signal transduction that directs cell growth and many other physiological processes, and whose activation is the final downstream component of various signal transduction pathways, including the *JAK-STAT* pathway. As the cytokine milieu described in PV shares significant similarities to that which promotes histiocyte recruitment, activation, and enhanced survival in ECD lesions,^5,8^ it is tempting to speculate that the pro-inflammatory effects of the *JAK2*V617F mutation may have contributed to the pathogenesis of ECD in our patient. It is important to remember that mutations leading to *JAK-STAT* pathway activation have been previously described in solid tumors such as head and neck squamous cell carcinomas, hepatitis B associated hepatocellular carcinomas, gastric adenocarcinomas, prostate cancer, nonsmall cell lung cancer, and glioblastomas.^18^

Low-dose aspirin and phlebotomies can be effectively and safely used to treat PV in low-risk patients, whereas hydroxyurea is usually added when treating those at high risk.^19^ Another treatment option is IFN-alpha, which can reduce the mutant clone,^20,21^ control constitutional and microvascular symptoms, and prevent thrombo-hemorrhagic complications in most cases. Furthermore, targeted therapies (mainly *JAK1/2* inhibitors) are now available and have led to promising results especially in patients who are resistant or intolerant to hydroxyurea.^22^

ECD therapy is recommended for all patients at the time of diagnosis, except those with minimally symptomatic disease. The possible therapeutic options include IFN-alpha-2a, pegylated IFN-alpha, corticosteroids, and imatinib, but no standard treatment regimen has yet been established.^1^

Interestingly, it has been reported that treatment with the *BRAF* inhibitor vemurafenib can lead to unprecedented clinical and radiographic improvements in ECD patients bearing the *BRAF*V600E mutation, but it has so far only been used in a very small number of patients enrolled in clinical trials.^23,24^ Furthermore, as *BRAF* is the final downstream component of the *JAK-STAT* pathway, it can be speculated that *JAK1/2* inhibitors such as ruxolitinib could also be used to treat ECD.
